# Restoration of wild-type motility to flagellin-knockout *Escherichia coli* by varying promoter, copy number and induction strength in plasmid-based expression of flagellin

**DOI:** 10.1016/j.crbiot.2020.03.001

**Published:** 2020-11

**Authors:** Nicholas M. Thomson, Mark J. Pallen

**Affiliations:** Quadram Institute Bioscience, Norwich Research Park, Norwich NR4 7UQ, United Kingdom

**Keywords:** Flagella, Motility, Promoter, Tuneable, Plasmid, Copy number

## Abstract

Flagellin is the major constituent of the flagellar filament and faithful restoration of wild-type motility to flagellin mutants may be beneficial for studies of flagellar biology and biotechnological exploitation of the flagellar system. However, gene complementation studies often fail to report whether true wild-type motility was restored by expressing flagellin from a plasmid. Therefore, we explored the restoration of motility by flagellin expressed from a variety of combinations of promoter, plasmid copy number and induction strength. Motility was only partially (~50%) restored using the tightly regulated rhamnose promoter due to weak flagellin gene expression, but wild-type motility was regained with the T5 promoter, which, although leaky, allowed titration of induction strength. The endogenous *E. coli* flagellin promoter also restored wild-type motility. However, flagellin gene transcription levels increased 3.1–27.9-fold when wild-type motility was restored, indicating disturbances in the flagellar regulatory mechanisms. Motility was little affected by plasmid copy number when dependent on inducible promoters. However, plasmid copy number was important when expression was controlled by the native *E. coli* flagellin promoter. Motility was poorly correlated with flagellin transcription levels, but strongly correlated with the amount of flagellin associated with the flagellar filament, suggesting that excess monomers are either not exported or not assembled into filaments. This study provides a useful reference for further studies of flagellar function and a simple blueprint for similar studies with other proteins.

## Highlights

• Restoration of motility to flagellin-knockout *E. coli* depends on choice of promoter.
• Plasmid copy number is important when using the natural flagellin promoter.
• For inducible promoters, induction strength is more important than copy number.
• Large increase in flagellin transcription but not flagella-associated protein.
• Plasmid-based expression interrupts flagellin expression control mechanisms.

## Graphical abstract

**Figure:**
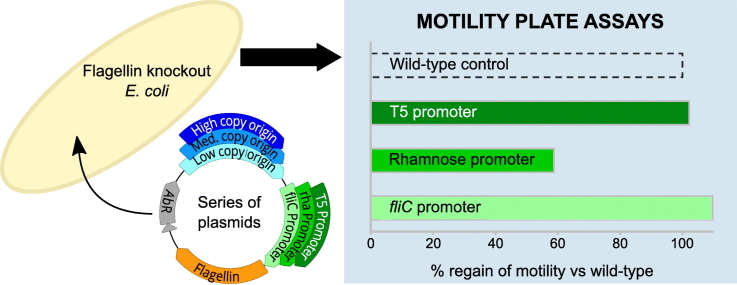

## 1 Introduction

Restoration of gene function in a knockout strain by *trans*-complementation is widely used to link genes to phenotypes. Similarly, expression of an engineered variant of a protein from a plasmid is widely used for biotechnological purposes. However, plasmid-based expression can disassociate a gene from its wild-type promoter and other regulatory influences and can lead to changes in gene copy number; all of which may lead to unwanted changes in gene expression and phenotype. This is a particular problem in synthetic biology, which relies on well-characterised, predictable gene expression when multiple genetic parts are joined to create new pathways, networks and products.

The flagellar filament in *Escherichia coli* is primarily responsible for swimming motility, but has also been implicated in interactions with host cells and the immune system (Chaban et al., 2015; Hajam et al., 2017). The filament is composed of multiple subunits of a single protein, flagellin (FliC), which travel in a semi-folded state through the hollow filament to assemble at the distal tip (Macnab, 2003). *E. coli* flagella are arranged peritrichously: with multiple flagella randomly distributed on the cell surface. Much of the elucidation of the genetic regulation, structure and function of flagellar systems has been carried out in *Salmonella enterica* Ser. Typhimurium, but the two species are closely related and the flagellar systems are similar in both (Apel and Surette, 2008; Chilcott and Hughes, 2000).

Around fifty different structural and regulatory genes are involved in flagellar synthesis. They are divided into three classes depending on the timing of their expression and are expressed in a gene synthesis cascade in response to global gene regulators such as heat shock proteins (Shi et al., 1992), the pleiotropic response regulator, OmpR (Shi et al., 1992), and the DNA-binding protein H-NS (Bertin et al., 1994). Gene class 1 contains a single operon, *flhDC*, known as the master operon of motility, transcription of which is controlled by sigma factor 70 (Yanagihara et al., 1999). The master operon activates the class 2 genes, which mostly encode the proteins of the hook-basal body (HBB) that anchors the flagellum in the cell membrane. Class 2 also includes four regulatory proteins: FliZ and FliT positively and negatively regulate structural genes, respectively, to ensure balanced expression (Kutsukake et al., 1999). FliA (sigma factor 28) initiates transcription of the class 3 genes. Meanwhile, FlgM is an anti-sigma factor, which binds to FliA and prevents it from activating gene expression (Chadsey et al., 1998; Kutsukake and Iino, 1994). FlgM-mediated repression of FliA action is released when an early class 3 protein, FliK, reaches sufficient quantities to direct the secretion of FlgM through the pore in the HBB and out of the cell (Karlinsey et al., 2000; Kutsukake, 1994). The removal of FlgM releases FliA to activate the switch to class 3 gene expression but can only take place following complete construction of the HBB, thus modulating the timing of the various components of the flagellum. Class 3 genes include the components of the flagellar filament, including FliC, chemotaxis-associated genes, and the HBB-associated motor proteins that drive flagellar rotation (Fitzgerald et al., 2014).

Interest has focused on engineering flagellin to display protein domains in the solvent-exposed regions for biotechnological purposes (Ezaki et al., 1998; Westerlund-Wikström, 2000; Yang et al., 2016). This typically requires wild-type levels of motility to be restored to a flagellin-knockout mutant by expressing FliC from a plasmid. However, when this has been attempted in previous studies, flagellar motility in the complemented strain has either not been compared to that in an isogenic wild-type strain (Kuwajima, 1988a, Kuwajima, 1988b; Mahajan et al., 2009), or where they have been compared, the complemented strain shows reduced motility (Lane et al., 2005). A further advantageous requirement might also be the ability to turn off or modulate the level of motility by controlling FliC expression with an inducible, tuneable promoter. We therefore sought to establish a baseline for effective restoration of wild-type motility and to explore the scope for fine-tuning levels of motility in *fliC*-deficient *E. coli* by comparing expression of flagellin from a variety of promoters and plasmids. Although our investigation focussed on motility, the general approach is easy to adopt and strongly recommended for any plasmid-based complementation system to ensure reliable interpretation of experimental results.

## 2 Material and methods

### 2.1 Bacterial strains, plasmids, and growth conditions

The bacterial strains and plasmids used in this study are summarised in Table 1. *E. coli* strain AW405 was a gift from Howard Berg (Harvard University). AW405 is resistant to streptomycin due to a *rpsL136* chromosomal mutation. Therefore, streptomycin (100 μg.mL^−1^) was included in all AW405 and AW405Δ*fliC* cultures as a precaution against contamination. Antibiotics were also added at the following concentrations as required for plasmid maintenance and selection (Table 1): carbenicillin (100 μg.mL^−1^), kanamycin (50 μg.mL^−1^), chloramphenicol (30 μg.mL^−1^). Unless otherwise specified, cultures were grown in 5 mL Miller's Luria-Bertani (LB) medium in 30 mL polystyrene, screw-capped universal tubes, at 37 °C and shaking at 200 rpm. When growth on solid medium was required, agar (1.5%) was added to the LB medium and plates were incubated statically at 37 °C.

**Table 1:** Bacterial strains and plasmids used in this study.

Name	Genotype/Features	Antibiotics	Source/ref.
*Bacterial strains*			
DH5α	F^−^, *Φ80lacZΔM15, Δ(lacZYA-argF), U169, recA, endA1, hsdR17(r*_*k*_^*−*^*,m*_*k*_^*+*^*), phoA, supE44, thi-1, gyrA96, relA1, λ*^*−*^	None	Invitrogen cat. No. 18265017
AW405	WT for motility and chemotaxis F^−^, *thr1, araC14, leuB6, fhuA31, lacY1, tsx-78, glnX44, galK2, galT22, λ, hisG4, rfbC1, mgl-51, rpsL136, xylA5, mtl-1, thiE1*	Streptomycin	(Turner et al., 2012)
AW405Δ*fliC*	As for AW405 but with *fliC* deleted; motility^−^	Streptomycin	This study

*Plasmids*			
pDOC-K	Donor plasmid for gene doctoring, with *sacB* gene for counterselection of successful transformants on sucrose medium	Kanamycin, Carbenicillin	(Lee et al., 2009)
pACBSCE	Helper plasmid for gene doctoring	Chloramphenicol	(Lee et al., 2009)
pCP20	Temperature-sensitive helper plasmid for *kanR* removal by FLP recombinase	Carbenicillin	(Lee et al., 2009)
pBb(RK2)1 k-GFPuv	PCR template for RK2 replication origin	Kanamycin	Addgene #106394, (Cook et al., 2018)
pJ211_WT_med	Expression plasmid for FliC *fliC* promoter; p15a (medium copy-number) replication origin	Kanamycin	ATUM custom order
pJ291_WT_med	Expression plasmid for FliC T5 promoter; p15a replication origin	Kanamycin	ATUM custom order
pD441_WT_hi	Expression plasmid for FliC T5 promoter; pUC (high copy-number) replication origin	Kanamycin	ATUM custom order
pJ211_WT_low	pJ211_WT_med derivative *fliC* promoter; RK2 (low copy-number) replication origin	Kanamycin	This study
pJ211_WT_hi	pJ211_WT_med derivative *fliC* promoter; pUC origin	Kanamycin	This study
pJ211_rha_WT_low	pJ211_WT_med derivative *rha* promoter; RK2 origin	Kanamycin	This study
pJ211_rha_WT_med	pJ211_WT_med derivative *rha* promoter; p15a origin	Kanamycin	This study
pJ211_rha_WT_hi	pJ211_WT_med derivative *rha* promoter; pUC origin	Kanamycin	This study
pJ291_WT_low	pJ291_WT_med derivative T5 promoter; RK2 origin	Kanamycin	This study

### 2.2 Selecting combinations of promoters and replication origins

The expression plasmids were based on pre-existing laboratory stocks of pJ211_WT_med, pJ291_WT_med, and pD441_WT_hi, all of which were custom-made by ATUM (Newark, CA, U.S.A.). These three plasmids all share the same backbone sequence, differing only in the promoter (and promotion inhibitor, in the case of the T5 promoter) and origin of replication. Each of the starting plasmids contained a copy of *fliC* from *E. coli* AW405 under control of either its natural promoter (pJ211) or the IPTG-inducible T5 promoter (pJ291 and pD441), and with either the medium copy-number p15a replication origin (med) or the high copy-number pUC origin (hi). To most closely replicate the low gene dosage of chromosome-encoded genes, we also selected the RK2 replication origin, which has previously been shown to consistently maintain plasmids with low copy numbers of ~5 per cell (Durland and Helinski, 1990; Jahn et al., 2016). The expressed FliC protein sequence is identical to that of the *E. coli* reference strain MG1655 (Uniprot entry P04949) belonging to H-serotype 48, containing 498 amino acids and with a molecular weight of 51.3 kDa.

The two promoters on the set of starting plasmids provided for either endogenously controlled or inducible expression. However, since *lac*-based promoters including T5 are not truly tuneable (Marschall et al., 2017), we also chose to include the rhamnose promoter to investigate whether tuneability of expression levels on the individual cell level would have a different effect on motility to the all-or-nothing response of the T5 promoter. We therefore constructed a set of nine plasmids with all combinations of three promoters and three replication origins.

### 2.3 Plasmid construction

All cloning operations were performed using *E. coli* DH5α as the host strain. Plasmids were purified when necessary using a NucleoSpin Plasmid Miniprep kit (Macherey-Nagel, Düren, Germany). In the first stage of cloning, the *fliC* promoter was replaced with the rhamnose promoter in pJ211_WT_med. The plasmid was digested with *Sal*I and *Nco*I and the linearized backbone was recovered by agarose gel purification. A gBlock containing the rhamnose promoter (from BioBrick BBa_K914003) and *E. coli rhaBAD* ribosome binding site together with SalI and NcoI restriction sites (Table 2) was purchased (IDT, Leuven, Belgium), digested and ligated to the backbone. This yielded pJ211_rha_WT_med.

**Table 2:** PCR primers and synthesised gene fragments used in the construction of plasmids for this study. Underlined sequences indicate restriction sites. Italic sequences indicate overlap regions for Gibson assembly. Complete sequences for each plasmid, including details of the primer binding sites, promoters, ribosome binding sites and replication origins are available online from Addgene under accession numbers 128848 – 128853 and 128855 – 128857.

Name	Sequence
*PCR primers*	
K12-N_5p_Eco_F	ATATATGAATTCCTGAATTGCGCAAAGTTTACG
K12-N_5p_Bam_R	ATATATGGATCCATTGCAAGTCGTTGATTACGT
K12-N_3p_Xho_F	ATATATCTCGAGGATTAACTGAGACTGACGG
K12_3p_Nhe_R2	ATATATGCTAGCTAAATTCCAGGCAGAAAAAAAC
RK2ori_Nhe_F	ATATATGCTAGCGCAGAGCCATGTAGGG
RK2ori_Hin_R	ATATATAAGCTTCCAGGCATCAAATAAAACGA
372F	CGATCGCCGTTTTCGTATCG
CC1	CTTAAGTTCTACGTGTTCCGC
2836R	TGTAGGGCGTCATAGCGTTC
CC2	CATGCTGGAGTTCTTCGCC
idnT_F	CTGTTTAGCGAAGAGGAGATGC
idnT_R	ACAAACGGCGGCGATAGC
hcaT_F	GCTGCTCGGCTTTCTCATCC
hcaT_R	CCAACCACGCAGACCAACC
cysG_F	TTGTCGGCGGTGGTGATGTC
cysG_R	ATGCGGTGAACTGTGGAATAAACG
fliC192_F	GGCCCGTAACGCCAACGACG
fliC289_R	CCGTCAGTTCACGCACACGC
1F	*CTACATGGCTCTGCGCTAGC*GCTGAGGTCCCGCAGC
4573R	*TATTTGATGCCTGGAAGCTT*CACTGCCCGCTTTCCAGT
G_Nhe_F	GCTAGCGCAGAGCCATGTAGGG
G_Hin_R	AAGCTTCCAGGCATCAAATAAAACGA

*Synthesised gene fragments*	
rhaBAD promoter + RBS	ATATATGTCGACCACCACAATTCAGCAAATTGTGAACATCATCACGTTCATCTTTCCCTGGTTGCCAATGGCCCATTTTCCTGTCAGTAACGAGAAGGTCGCGTATTCAGGCGCTTTTTAGACTGGTCGTAATGAAATTCAGCAGGATCACACCATGGATATAT
pUC origin	ATATATAAGCTTGAGATCCTTTTTTTCTGCGCGTAATCTGCTGCTTGCAAACAAAAAAACCACCGCTACCAGCGGTGGTTTGTTTGCCGGATCAAGAGCTACCAACTCTTTTTCCGAAGGTAACTGGCTTCAGCAGAGCGCAGATACCAAATACTGTTCTTCTAGTGTAGCCGTAGTTAGGCCACCACTTCAAGAACTCTGTAGCACCGCCTACATACCTCGCTCTGCTAATCCTGTTACCAGTGGCTGCTGCCAGTGGCGATAAGTCGTGTCTTACCGGGTTGGACTCAAGACGATAGTTACCGGATAAGGCGCAGCGGTCGGGCTGAACGGGGGGTTCGTGCACACAGCCCAGCTTGGAGCGAACGACCTACACCGAACTGAGATACCTACAGCGTGAGCTATGAGAAAGCGCCACGCTTCCCGAAGGGAGAAAGGCGGACAGGTATCCGGTAAGCGGCAGGGTCGGAACAGGAGAGCGCACGAGGGAGCTTCCAGGGGGAAACGCCTGGTATCTTTATAGTCCTGTCGGGTTTCGCCACCTCTGACTTGAGCGTCGATTTTTGTGATGCTCGTCAGGGGGGCGGAGCCTATGGAAAGCTAGCATATAT

In the second stage, the p15a replication origin was replaced by RK2 in each of the three medium copy plasmid variants. For plasmids with the pJ211 backbone, RK2 was amplified from pBb(RK2)1 k-GFPuv (Cook et al., 2018), which was a gift from Brian Pfleger (University of Wisconsin-Madison), using Q5 DNA polymerase (NEB, Hitchin, U.K.) and primers RK2ori_Nhe_F and RK2ori_Hin_R (Table 2). The product was digested with *Nhe*I and *Hin*dIII, gel purified and ligated into the gel purified plasmid backbones, which had been digested with the same enzymes. This yielded pJ211_WT_low and pJ211_rha_WT_low. The pJ291 backbone is missing the NheI and HindIII recognition sites. Therefore, pJ291_WT_low was constructed by Gibson Assembly, which was designed to introduce the sites into the plasmid for future use. The backbone was amplified using primers 1F and 4573R and the RK2 origin was amplified using primers G_Nhe_F and G_Hin_R (Table 2). Both fragments were gel purified and then joined following the manufacturer's instructions for the Gibson Assembly kit (NEB, Hitchin, U.K.).

Finally, pJ211_WT_med and pJ211_rha_WT_med were used for replacement of the p15a replication origin by the pUC origin. This proceeded as for insertion of RK2, except the pUC origin sequence was purchased as a gBlock (IDT, Leuven, Belgium) containing NheI and HindIII restriction sites (Table 2). The resulting plasmids were pJ211_WT_hi and pJ211_rha_WT_hi. All nine plasmid sequences were verified by Illumina shotgun sequencing in the DH5α host cells and mapping reads to the expected plasmid sequence. Complete sequences for each plasmid, including details of the primer binding sites, promoters, ribosome binding sites and replication origins are available online from Addgene under accession numbers 128848 – 128853 and 128855 – 128857. The function of the promoter-flagellin combinations were verified by performing a motility assay on cells of AW405Δ*fliC* transformed with each plasmid. Motile cells from each assay were recovered and grown overnight to produce glycerol stocks, which were used for all other experiments.

### 2.4 Creation of a *fliC* knockout strain

A *fliC* deletion mutant of AW405 was created using the gene doctoring system for chromosomal gene editing (Lee et al., 2009). Plasmids pDOC-K, pACBSCE and pCP20 were gifts from Stephen Busby (University of Birmingham). Briefly, homologous regions of 200 bp upstream and downstream of the *fliC* gene were cloned into pDOC-K using EcoRI/BamHI (upstream) and XhoI/Nhe (downstream) restriction sites. The inserted sequences were acquired from colony PCR reactions with AW405 as the template, Q5 DNA polymerase and primers K12-N_5p_Eco_F, K12-N_5p_Bam_R (upstream), K12-N_3p_Xho_F, and K12_3p_Nhe_R2 (downstream) (Table 2), followed by agarose gel purification using a NucleoSpin Gel and PCR Cleanup kit (Macherey-Nagel, Düren, Germany).

The plasmids pDOC-K (containing the homologous regions) and pACBSCE were then inserted into AW405 by co-electroporation and cells containing both plasmids were selected for on LB agar containing kanamycin, carbenicillin and chloramphenicol. A single colony was picked, grown to late exponential phase in LB + antibiotics (0.5 mL), washed free of antibiotics in 0.1× LB and incubated at 37 °C for 1 h in the same volume of 0.1× LB containing 0.5% L-arabinose to induce expression of the λ-Red recombinase. Successful recombinants were selected for by plating on LB agar with 5% sucrose and screened for loss of both plasmids by checking susceptibility to carbenicillin and chloramphenicol. Deletion of the *fliC* gene was confirmed by colony PCR using OneTaq DNA polymerase (NEB, Hitchin, U.K.) and primers 372F, CC1 (upstream fragment), 2836R and CC2 (downstream fragment).

The *kanR* resistance cassette was removed by transformation with pCP20 and growth of carbenicillin resistant colonies in LB at 30 °C overnight. The temperature-sensitive pCP20 was then removed by growth at 37 °C without antibiotics. Colonies were finally checked for susceptibility to kanamycin, carbenicillin and chloramphenicol, and verified by colony PCR with primers 372F and 2836R, and shotgun sequencing.

### 2.5 Plasmid and genome sequence analysis

Genomic DNA was purified using a FastDNA Spin Kit for feces (MP Bio, Santa Ana, CA, U.S.A.) according to the manufacturer's instructions, except that the final elution was in 200 μL of DNAse-free water rather than 60 μL. Plasmids were purified with a NucleoSpin Plasmid Miniprep kit. The DNA was quantified using a Quant-iT dsDNA high sensitivity assay kit (Thermo Fisher, Waltham, MA, U.S.A.) and normalised to 0.2 ng.μL^−1^ in 10 mM Tris-HCl. Sequencing libraries were prepared with the Nextera XT DNA Library Prep kit (Illumina, San Diego, CA, U.S.A.). Libraries were quantified using the Quant-iT dsDNA high sensitivity assay kit. Genome samples were pooled in equal quantities, while plasmid samples were first pooled together and the whole plasmid pool was added to the genomic pool at one-tenth the quantity of a genomic sample. The final pool was then run at a final concentration of 1.8 pM on an Illumina NextSeq 500 instrument using a mid-output sequencing kit for 150 bp paired-end reads. Sequences were quality checked by FastQC v0.11.7 and trimmed with Trimmomatic v0.36 with a minimum read length of 40 bp and a sliding window of 4 bp with average quality of 15. The reads were then mapped against the expected sequence for each sample in Geneious R11.1 with the Geneious mapper and default settings.

### 2.6 Motility assays

Motility assays were performed on motility agar (MA) plates, which consisted of (per L) Difco Lab-Lemco powder (10 g), Difco meat extract (3 g), NaCl (5 g) and agar (3 g). 30 mL of medium was solidified in a 90 mm triple-vented Petri dish for each measurement. Kanamycin, L-rhamnose (Sigma-Aldrich, Gillingham, U.K.) and Isopropyl β-D-1-thiogalactopyranoside (IPTG; Sigma-Aldrich, Gillingham, U.K.) were included at the specified concentrations whenever necessary. A master plate of each strain was initially prepared by stabbing from a glycerol stock into the centre of a MA plate and incubating at 30 °C overnight. Subsequent inoculations were made from the outer edge of the motile disk on the master plate.

Three biological replicates were grown for each strain and three technical replicates were inoculated from each culture. The cultures were grown in motility broth (MB; as for MA but omitting the agar) at 30 °C overnight, then normalised to OD_600_ = 0.5. 1 μL of normalised culture was applied to the centre of a MA plate by stabbing a 10 μL pipette tip into the agar, taking care not to reach through to the bottom, and ejecting the inoculum. Plates were then incubated at 30 °C for 24 h and motility was quantified by measuring the diameter of the motile disk. All assays from each promoter type were performed using the same batch of MA, and each biological replicate was performed on the same day. Due to the volume of media required it was not possible to perform all assays simultaneously, and this gave rise to some batch-to-batch variation. Therefore, an AW405 wild-type control was performed in triplicate alongside each set of plates, and the final motility scores were scaled according to the mean of all wild-type assays (28.88 mm) to allow accurate comparisons between promoters.

### 2.7 Reverse transcriptase-quantitative polymerase chain reaction

Flagellin may be present in the cytoplasm (either as soluble or insoluble protein), assembled into flagellar filaments, or diluted in the external medium as unpolymerised monomers and fragments of broken filaments. Therefore, obtaining an accurate measure of total flagellin protein is challenging. However, promoter induction strength and plasmid copy number are expected to modulate flagellin expression at the transcriptional level, so we measured the levels of *fliC* transcription relative to the wild-type strain for each plasmid construct using reverse transcriptase-quantitative polymerase chain reaction (RT-qPCR). We attempted to recover RNA directly from the motile cultures on plates but a combination of relatively low available cell numbers and agar inhibiting cell lysis meant that we were unable to recover sufficient RNA. Therefore, we extracted RNA from liquid MB cultures grown in Petri dishes to maintain conditions as close as possible to the motility assays.

Starter cultures (in triplicate) were grown as for motility assays and then normalised to OD_600_ = 0.1. A 1 μL aliquot of each normalised culture was then transferred to the centre of each plate containing 30 mL of MB + streptomycin, kanamycin, L-rhamnose and IPTG as necessary and the cultures were grown for 24 h at 30 °C statically. For the inducible promoters, we included the concentration of inducer that gave motility closest to the wild-type as reported in Section 3.1, i.e. 0.5% rhamnose for the *rha* promoter and 50, 500, and 250 μM IPTG for the T5 promoter with low, medium and high copy origins, respectively. Positive and negative control cultures consisting of AW405 and AW405Δ*fliC*, respectively, were grown in the same way. RNA was recovered from 20 mL of the cultures using an SV Total RNA Isolation kit (Promega, Madison, WI, U.S.A.). To ensure complete removal of DNA, the samples were then treated with a DNAse Max kit (Qiagen, Venlo, Netherlands). The RNA concentration was determined by Qubit RNA high sensitivity assay (Invitrogen, Inchinnan, U.K.) and integrity checked by TapeStation RNA assay (Agilent, Stockport, U.K.), then adjusted to 3.75 ng.μL^−1^ with nuclease-free water.

Primers were designed in Geneious Prime 2019. *fliC* primers were fliC_192_F and fliC_289_R (Table 2). The endogenous controls were *idnT, hcaT* and *cysG* (Zhou et al., 2011) and used primers idnT_F, idnT_R, hcaT_F, hcaT_R, cysG_F and cysG_R (Table 2). Reactions were set up in 384-well microplates (4titude, Dorking, U.K.) and consisted of 3 μL PrecisionPLUS OneStep RT-qPCR Master Mix with low ROX and SYBR green (Primer Design, Chandler's Ford, U.K.), 0.3 μM final concentration of each primer, 2 μL normalised RNA solution and water to a final volume of 6 μL. Calibration curves for each target were constructed by triplicate reactions of seven 10-fold serial dilutions of purified AW405 gDNA (starting from 40 ng.μL^−1^). Reactions were run with 3 technical replicates each on a ViiA7 Real-time PCR system (Thermo Fisher, Waltham, MA, U.S.A.) programmed as follows: 55 °C for 10 min, 95 °C for 2 min, 45 cycles of 95 °C for 10 s and 68 °C for 60 s, then a melt curve analysis using 95 °C for 15 s, 60 °C for 60 s and ramping at 0.05 °C.*sec*^−1^ to 95 °C. The elevated extension temperature was used to minimise the formation of primer dimers. Data were analysed in QuantStudio v.1.3 (Thermo Fisher, Waltham, MA, U.S.A.) using the relative standard curve method.

### 2.8 SDS-polyacrylamide gel electrophoresis and densitometry

The same MB liquid cultures were used as for RT-qPCR. Cells in 5 mL aliquots were pelleted by centrifugation at 3000 ×*g* for 10 min. The culture with the lowest OD_600_ was resuspended in 200 μL phosphate buffered saline by vigorous vortexing and the other cultures were resuspended in a volume to give an equal cell density. Flagella were depolymerised at 65 °C for 5 min in a water bath and the cells were removed by centrifugation at 17,000 ×*g* for 10 min. 25 μL of each supernatant was mixed with an equal volume of 2× Novex Tris-Glycine SDS Sample Buffer (Invitrogen, Inchinnan, U.K.) and 40 μL of the mixture was run on an 8% Novex tris-glycine SDS-PAGE gel for 50 min at 200 V and stained using InstantBlue Coomassie Stain (Expedeon, Cambridge, U.K.). The gels were photographed and the density of FliC bands compared using the gel analyser function of ImageJ v1.52n. The 55 kDa band from the Color Prestained Protein Standard, Broad Range (NEB, Hitchin, U.K.) was used to normalise for staining differences between gels.

## 3 Results

### 3.1 Motility assays

**Fig. 1:**
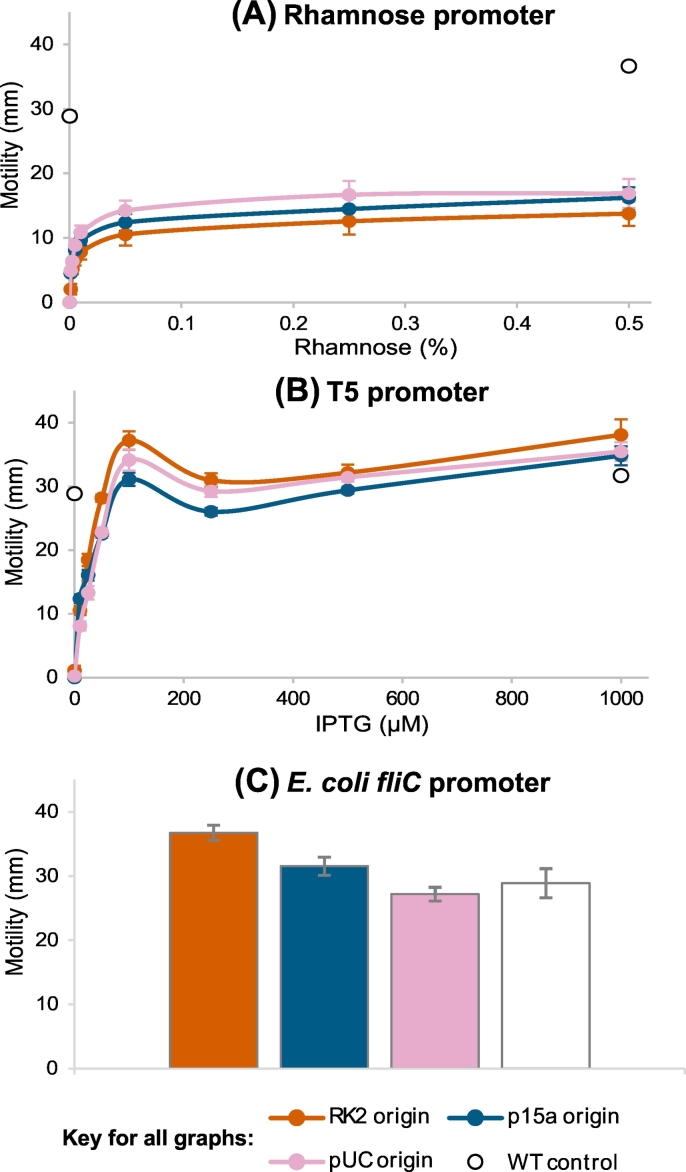
Variation in motility for expression of FliC from low (RK2), medium (p15a) and high (pUC) copy number plasmids with different combinations of promoter and induction strength. Motility was assayed by incubating inoculations for 24 h at 30 °C on soft motility agar plates and measured as the diameter of the motile swarm. (A) FliC under control of the rhamnose promoter, induced by a range of rhamnose concentrations. (B) FliC under control of the T5 promoter, induced by a range of IPTG concentrations. (C) FliC expressed from its natural promoter, which is not chemically induced. Wild-type strain AW405, with FliC expressed from the chromosome was assayed as a control. Results are the means of at least 3 biological repeats, each with 3 technical repeats ± SEM.

We transformed each of the nine variant plasmids into *E. coli* AW405Δ*fliC* and conducted motility assays in 0.3% motility agar plates. The rhamnose promoter plasmids showed no motility in the absence of rhamnose (Fig. 1 (A)). Motility increased in proportion to rhamnose concentration up to approximately 0.01% with a small further increase for higher concentrations, and reached a plateau at approximately 0.1%. The low copy number plasmid (RK2 origin) consistently gave the lowest motility, followed by the medium copy plasmid (p15a origin) and the high copy number plasmid (pUC origin) gave the highest motilities. However, the differences between plasmids were small. In all cases, motility was significantly lower than for the WT control (*t*-test; *p* < 10^−4^).

**Fig. 2:**
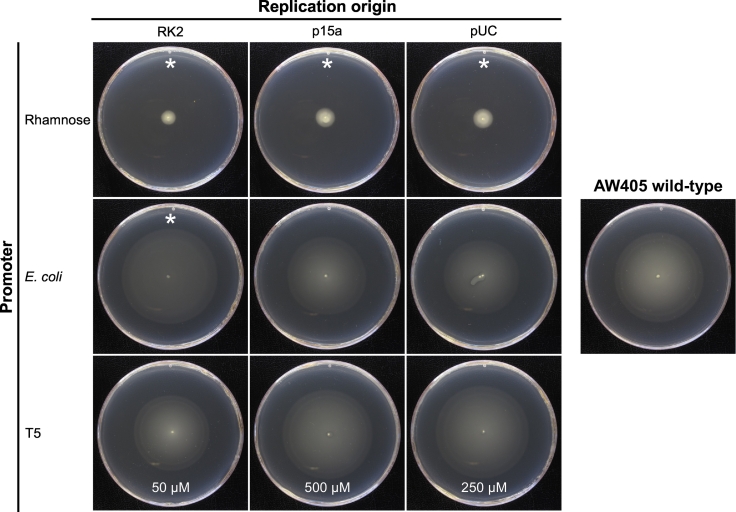
Representative motility agar plates with the conditions that most closely restored wild-type motility for each tested plasmid. All plates were incubated at 30 °C for 24 h. The results with the rhamnose promoter were all achieved with 0.5% rhamnose. Concentrations of IPTG that gave motility closest to wild-type are given at the bottom of the corresponding plates. * = mean motility under these conditions was significantly different to wild-type (*t*-test; *p* < 10^−4^).

The T5 promoter also gave reasonably tight control over motility, although in a few cases leaky expression led to low levels of motility in the absence of IPTG. Motility increased with IPTG concentration up to approximately 100 μM (Fig. 1 (B)). Interestingly, for each plasmid, 100 μM IPTG resulted in a local maximum motility, with lower motility at 250 mM. The motility then gradually increased again up to 1000 μM IPTG. The differences in motility between plasmids were small, with a consistent trend of p15a < pUC < RK2. Motilities peaked at above the level of the control, especially with the low copy number plasmid. However, for each plasmid it was possible to replicate wild-type motility by adjusting the concentration of IPTG (Fig. 2).

The *E. coli fliC* promoter is controlled by the endogenous flagellar expression cascade (Lemke et al., 2009) and so is not regulatable. There was an inverse correlation between plasmid copy number and observed motility (Fig. 1 (C)). The high copy number (5.9% decrease) and medium copy number (9.2% increase) plasmids replicated wild-type motility (*t*-test; *p* > .1; Fig. 2), while motility from the low copy number plasmid showed a significant increase of 27.3% over the wild-type (t-test; *p* < 10^−4^).

### 3.2 Flagellin production

**Fig. 3:**
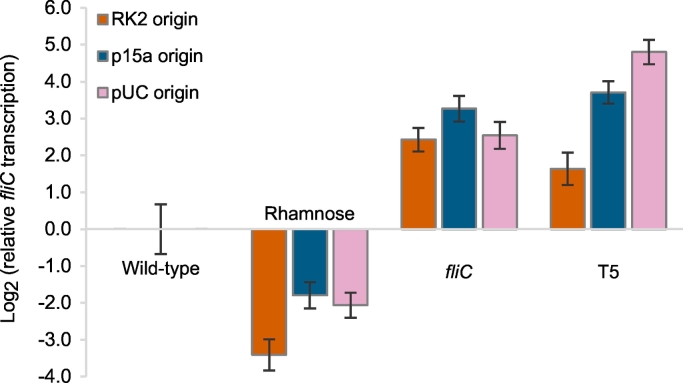
Log_2_-transformed FliC transcription levels at late-exponential phase relative to wild-type AW405, as measured by RT-qPCR. A *fliC* knockout strain did not give amplification of the target *fliC* fragment. Results are the means of 3 biological replicates each with 3 technical replicates ±1 standard deviation.

We recovered RNA from triplicate cultures of each strain, including the wild-type and AW405Δ*fliC* controls. AW405Δ*fliC* did not show amplification of a *fliC* target fragment but had similar levels of the endogenous controls. The *cysG* endogenous control produced primer dimers, as indicated by a second peak on the melting curve. Expression of *cysG* between samples also varied the most of the endogenous controls. Therefore, we excluded *cysG* from the analysis and normalised *fliC* expression against *idnT* and *hcaT*. All of the nine plasmids gave expression levels that differed significantly from the wild-type (*t*-test; *p* = .002 to 2.6 × 10^−5^; Fig. 3). The *fliC* and T5 promoters both increased transcription (relative quantity (RQ) range 3.11 to 27.92) irrespective of plasmid copy number, while the rhamnose promoter decreased transcription (RQ range 0.09 to 0.24). Triplicate technical repeats of each reaction showed very low variance but there was moderate variance between biological replicates. This is suggestive of stochasticity in *fliC* expression at the level of individual cells and might also reflect variation in *fliC* transcription at slightly different cell densities.

There was no overall trend linking transcription levels with plasmid copy number (Fig. 3), although the T5 promoter did show greater transcription as the plasmid copy number increased, with significant differences between each plasmid (*t*-test; *p* < .005). The induction strength was tuned for each strain to reinstate wild-type motility, so it might be expected that *fliC* transcription levels would in fact be similar. However, the motility results were very similar for all IPTG concentrations above 50 μM (Fig. 1) suggesting saturation of the flagella assembly mechanism. Only the rhamnose promoter showed the same trend between copy numbers for motility and FliC expression. For the *fliC* and T5 promoters, low copy number plasmids showed transcription levels most like the wild-type. For the rhamnose promoter, the medium and high copy number plasmids had the same level of expression (*t*-test; *p* > .5) and were closest to wild-type. This might reflect the relative weakness of the rhamnose promoter. Overall, reduced expression of FliC from the weak rhamnose promoter correlated well with the observed reduced motility, while increased transcription levels from the other promoters did not translate to increased motility.

### 3.3 Quantification of filament-associated flagellin

**Fig. 4:**
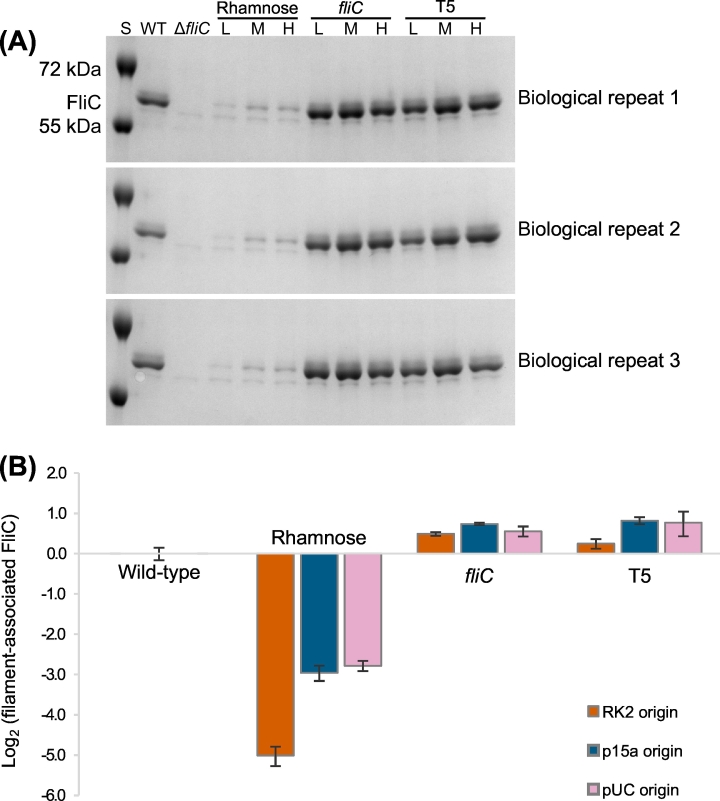
Densitometry analysis of levels of filament-associated flagellin. (A) SDS-PAGE gels for each biological replicate, from which densitometry measurements were taken. Flagella were depolymerised by heat treatment and the monomers were separated from cells and denatured before loading onto gels with their quantities normalised by cell density. S = molecular weight standards; L = low copy number (RK2) plasmid; M = medium copy number (p15a) plasmid; H = high copy number (pUC) plasmid. (B) Log2-transformed levels of filament-associated flagellin in each strain relative to wild-type AW405. A *fliC* knockout strain gave a small signal by densitometry that was not distinguishable from background noise and other proteins, and so is not shown here. Results are the means of 3 biological replicates ±1 standard deviation.

Only flagellin that is correctly assembled into a flagellar filament can contribute towards cell motility. Therefore, in addition to quantifying the total transcription of *fliC*, we also recovered the filament-associated protein by heat depolymerisation and compared the concentrations between each strain by densitometry of SDS-PAGE bands. The flagellin protein was easily identified as a densely-stained band with apparent molecular weight of 60 kDa (Fig. 4 (A)), slightly larger than the weight predicted from the sequence (Kondoh and Hotani, 1974).

As for *fliC* transcription, plasmid-based flagellin expression gave significantly different levels of filament-associated flagellin for all (t-test; *p* = .046 to 1.5 × 10^−4^) except the T5 promoter/low copy number combination (*p* = .13, Fig. 4 (B)). The *fliC* and T5 promoters increased filament-associated FliC, although the magnitude of the increase was much smaller than for gene expression (RQ range 1.18 to 1.76), while the rhamnose promoter decreased filament-associated FliC by a similar magnitude to gene expression (RQ range 0.03 to 0.15). The trends in protein abundance between plasmid copy numbers were remarkably similar for gene expression and filament-associated FliC. Overall, decreased transcription of *fliC* leads to less flagellin incorporated into flagellar filaments and lower motility, but increased transcription does not necessarily translate to larger quantities of filament-associated flagellin or higher motility.

## 4 Discussion

We compared the levels of *E. coli* flagellin expression, incorporation into flagellar filaments, and consequent motility in isogenic AW405Δ*fliC* strains containing a series of nine plasmids with different combinations of promoters and replication origins. Our aim was to faithfully reinstate wild-type motility to the *fliC* knockout mutant by tuning the expression of wild-type flagellin from the plasmids. The results presented here demonstrate that for a complex system such as the *E. coli* flagellum it can be difficult to predict the effects of manipulating the expression of a single component. Flagellin production and incorporation into flagellar filaments is the final part of a complex gene regulation cascade, involving >50 different structural proteins and transcription factors (Soutourina et al., 1999), of which we controlled the expression of only one gene directly. Since the multiple previous stages in the construction of the flagellum largely determine the magnitude of motility, it is expected that there will not be a simple, direct correlation between flagellin expression and motility.

Due to dosage effects, the low copy number plasmid should most faithfully reproduce the wild-type phenotype as it is most similar to the few copies of *fliC* that are likely to be present on the chromosome (note that during exponential growth, *E. coli* replicates the chromosome faster than cell division and so maintains multiple, rather than single, copies of most genes (Sobetzko et al., 2012)). We found that plasmid copy number had only a small effect in this regard, indicating that the choice of chassis plasmid for gene complementation is relatively unimportant. Surprisingly, our motility results indicate that choosing a medium or high copy number plasmid is slightly preferable to a low copy number when expression is driven by the *fliC* promoter. Our RT-qPCR results showed fairly stable levels of *fliC* transcription between plasmids, suggesting that the flagellar regulatory machinery remains functional to some extent when flagellin is expressed from a plasmid rather than the chromosome and can attenuate the effects of high gene copy number. The negative regulator of flagellar class 3 gene expression, FlgM is produced in greater quantities during class 3 gene expression (Ikebe et al., 1999), providing a negative feedback mechanism that may explain this finding. However, transcription levels still increased relative to the wild-type and to a greater extent than the filament-associated FliC, probably because the greater gene dosage reduced the efficacy of FlgM negative regulation.

The rhamnose and T5 promoters break the connection between *fliC* and the rest of the flagellar structural and regulatory components as they are not dependent on FliA and were induced throughout the experiment. With these promoters it is possible to tune cell motility based on flagellin production, from preventing motility in the absence of inducer to replicating wild-type motility with ~250 μM IPTG. Although inducer concentration was the dominant factor in determining motility, within each concentration bracket there was a small additional effect of plasmid copy number. Increasing plasmid copy number with the inducible promoters increased *fliC* transcription but only slightly increased the amount of filament-associated flagellin at the maximum induction strength. It would therefore appear that increasing flagellin production does not increase motility.

If regulation of the HBB remains the same as in the wild-type, continuous expression from the T5 promoter would cause a build-up of FliC that is not able to be secreted while the HBB is being formed. Even after formation of the HBB, FliC production would not be responsive to the FliA/FlgM negative-feedback mechanism and may still progress at a faster rate than can be accommodated by the secretion mechanism or the flagellar assembly process, leading to accumulation of FliC monomers either in the cytoplasm or extracellularly (Kutsukake and Ide, 1995). Together with gene dosage effects, this explains the much larger levels of *fliC* transcription from the T5 promoter, which do not result in more filament-associated FliC. The low motilities and low concentrations of filament-associated flagellin observed from the rhamnose promoter suggested that it is a relatively weak promoter and incapable of producing sufficient flagellin for wild-type motility. This is supported by the observation that higher copy number plasmids allowed slight increases in motility and gene expression with this promoter.

The choice of promoter and induction strength is crucial. While the rhamnose promoter allowed tight control of motility and was finely tuneable, filament-associated flagellin and motility remained significantly below wild-type under all tested conditions. On the other hand, both the natural *E. coli fliC* promoter and the IPTG-inducible T5 promoter were able to completely rescue the motility phenotype. From this, we would recommend use of the natural promoter for the gene of interest wherever possible because it will be responsive to endogenous control mechanisms. However, if manual control of timing or levels of expression are required, titration of induction strength from an inducible promoter such as T5 may be preferable.

It is notable that despite restoring wild-type motility with both the *fliC* and T5 promoters, we were unable to match the wild-type levels of gene expression. For most purposes, simply restoring motility is likely to be enough. However, this could be problematic for mechanistic studies of the flagellar system. The most predictable way to reinstate wild-type gene expression and motility may be to re-insert the gene into the chromosome, perhaps under control of an inducible promoter if control over gene expression would be useful. However, chromosomal manipulations are time-consuming, difficult to perform in high-throughput, often leave scar sequences from removal of a selection marker, and generally require the production of a plasmid containing the sequence to be inserted. Therefore, particularly for screening large numbers of genes or gene variants, it is far more convenient to express the desired gene from a plasmid in the first instance and to follow up with more detailed studies of chromosome-based expression if necessary.

The set of 9 expression plasmids constructed for this study may be useful tools for future similar studies with other proteins. The results here apply to motility in *E. coli* but should also be applicable to closely related Gram-negative bacteria, especially *S*. *enterica*. Considering the dominance of these species as model organisms for flagellar biology and of *E. coli* for synthetic biology, we expect the results will be relevant to numerous future studies. Gram-positive bacteria and those with different flagellation patterns have different regulatory mechanisms, which may respond differently to plasmid-based FliC expression (Kazmierczak and Hendrixson, 2013). In these cases, for similar studies with non-flagellar proteins or simply as general expression vectors it may be desirable to clone different genetic elements. Our plasmids can easily be modified to make use of different promoters by cloning alternative sequences between the *Sal*I and NcoI restriction sites, or alternative promoters between the NheI and HindIII restriction sites. Any gene of interest can also replace the *fliC* sequence by in-frame insertion between the NcoI and EcoRI restriction sites.

This study has provided useful guiding principles for the specific case of restoring motility in *E. coli*. We expect that this will facilitate further investigations into the structure and function of the flagellum and high-throughput biotechnological applications such as the development of flagellar display technology for large, globular protein domains. It should also serve as a reminder that simple optimisation and verification of protein expression strategies should be performed to separate the effects of the protein itself from convoluting factors from the expression system.

## CRediT authorship contribution statement

**Nicholas M. Thomson:** Conceptualization, Methodology, Validation, Formal analysis, Investigation, Writing - original draft, Writing - review & editing, Visualization. **Mark J. Pallen:** Conceptualization, Resources, Writing - original draft, Writing - review & editing, Supervision, Funding acquisition.

## Declaration of competing interest

We wish to confirm that there are no known conflicts of interest associated with this publication and there has been no significant financial support for this work that could have influenced its outcome.
